# Investigation of the Influence of Open-Die Forging Parameters on the Flow Kinetics of AZ91 Magnesium Alloy

**DOI:** 10.3390/ma14144010

**Published:** 2021-07-17

**Authors:** Grzegorz Banaszek, Teresa Bajor, Anna Kawałek, Tomasz Garstka

**Affiliations:** 1 Department of Materials Engineering, Faculty of Production Engineering and Materials Technology, Czestochowa University of Technology, 42-201 Czestochowa, Poland; grzegorz.banaszek@pcz.pl (G.B.); tomasz.garstka@pcz.pl (T.G.); 2 Departmet of Production Management, Faculty of Production Engineering and Materials Technology, Czestochowa University of Technology, 42-201 Czestochowa, Poland; anna.kawalek@pcz.pl

**Keywords:** magnesium alloy AZ91, physical modelling, open die forging, flat anvils, shaped anvils

## Abstract

This paper presents the results of numerical tests of the process of forging magnesium alloy ingots (AZ91) on a hydraulic press with the use of flat and proprietary shaped anvils. The analysis of the hydrostatic pressure distribution and the deformation intensity was carried out. It is one of the elements used for determining the assumptions for the technology of forging to obtain a semi-finished product from the AZ91 alloy with good strength properties. The aim of the research was to reduce the number of forging passes, which will shorten the operation time and reduce the product manufacturing costs. Numerical tests of the AZ91 magnesium alloy were carried out using commercial Forge^®^NxT software.

## 1. Introduction

The search for lightweight construction materials with favourable strength parameters, which has continued over several decades, has led scientists toward magnesium alloys [1,2,3]. The implemented environmental protection policy, the main goal of which is to reduce the weight of cars and fuel consumption, and to reduce the impact of greenhouse gases emitted by cars, is not without significance in this matter [4,5,6,7]. Magnesium alloys, being the lightest construction materials and showing good heat dissipation and vibration damping, are found to have more and more applications in the automotive industry. They are used to produce elements for operation at ambient temperatures, such as brackets, covers, or casings of modern cars [8,9,10,11]. The vast majority of products made of magnesium alloys are obtained mainly in extrusion and stamping processes and, less frequently, in rolling and forging processes.

However, forged products made of magnesium alloys deserve special attention due to their homogeneous microstructure and improved mechanical properties compared to elements made of cast alloys. Due to their properties, the products obtained in the forging process are also attractive to such industries as: shipbuilding, aviation, space, and electronics [12,13,14]. The unquestionable advantage of using open-die forging processes to deform magnesium alloys is the possibility of freely shaping the metal flow kinematics, including by controlling the shape and dimensional parameters of anvil working surfaces and the main parameters of extending operations, which is often impossible, e.g., in extrusion processes. It is also possible to forge magnesium alloys in differently-shaped anvils. The use of shaped anvils allows for the introduction of the intended nature of stresses and strains in the local areas of the forgings. Additionally, the advantage of this forging method is the reduction of the number of forging passes, which reduces the production costs of the final products [15].

The implementation of research in the field of designing forging technology requires a comprehensive approach to the research problem. A high variability of shaping parameters (such as temperature and sequence of operations) and technological procedures (such as material rotation and deformation by means of anvils during the forging elongation operation, values of the set reductions, relative feed values, deformation speed, and shape and dimensional parameters of the anvil working surfaces) makes it extremely difficult to obtain forgings from high-quality magnesium alloys with a homogeneous microstructure throughout the entire volume. Due to the hexagonal, compact, crystallographic structure, magnesium alloys have limited plasticity and poor deformability at ambient temperature. They are also characterised by a high anisotropy of mechanical and plastic properties arising during the given plastic deformation. Due to these factors, there is an increase in demand for a technology for the production of magnesium alloys with better strength properties. Proper implementation of the open-die forging process in flat and shaped anvils of selected magnesium alloys requires not only special forging equipment but, most of all, necessary specialist knowledge in this field. Undertaking the research into the theoretical analysis of plastic forming of the AZ91 magnesium alloy is one of the stages of designing the technology of manufacturing finished products with good mechanical properties [15,16,17,18,19].

## 2. Test Objective and Scope

The aim of the paper was to determine the distributions of hydrostatic pressure and strain intensity in AZ91 magnesium alloy rods, shaped by open forging in flat and shaped anvils, based on model tests of these technological processes. The use of shaped anvils will reduce the number of forging passes and, at the same time, reduce the cost of manufacturing the product.

Modelling of the forging process was performed using the Forge^®^NxT software commercial software (version 2.0) Transvalor S.A., Biot, Sophia Antipolis cedex, France based on FEM, which allows for the determination of changes in the value of hydrostatic pressure and strain intensity in the plastically processed metal at any stage of the forging elongation operation. Based on the analysis of the results of numerical tests, assumptions for the technology of producing magnesium alloy bars by the open-die forging method in flat and shaped anvils will be developed.

The obtained test results should contribute to the improvement of the mechanical properties of the finished products through the appropriate selection of technological parameters of the open-die forging process in flat and shaped anvils.

The ranges of deformation, deformation speed, and temperature changes during the theoretical research were assumed on the basis of the characteristics of forging machines such as: PHM 250 T Żywiec, Poland or PWH-250R, Poland used in real forging processes and on the basis of literature data [15,20].

## 3. Materials and Methods

The material chosen for the tests was magnesium alloy AZ91 with chemical compositions as given in Table 1 [15].

## 4. Determination of Rheological Properties of the Selected Magnesium Alloy

Knowledge of the characteristics describing the technological properties of the material is the basis for the correct conduct of the theoretical research and the design of new technological processes (or modification of existing ones). For each technological process of plastic working, a set of data that accurately describes the material’s susceptibility to plastic forming should be defined [15].

For plastic working processes, the basic feature characterising the material’s susceptibility to plastic forming is the flow stress σ**_p_** and limit strain ε_g_.

The flow stress σ_p_, i.e., the stress necessary to initiate and continue plastic flow of metal under uniaxial stress conditions, is a function of strain (ε), strain rate ($\dot{\varepsilon }$), temperature (T) and history of strain.

During hot plastic working, processes resulting from the plastic deformation mechanism, material hardening, and thermally activated processes, as well as time-dependent phenomena leading to material weakening, occur in the material structure. Thus, it is relatively difficult to determine the technological plasticity characteristics of the material for the conditions of hot plastic working [15].

The values of flow stress σ_p_ in computer programmes intended for modelling plastic working processes by the finite element method are determined on the basis of the assumed flow stress function. The flow stress is described by the dependence in the form of $\sigma_{p}=\left(\varepsilon ,\dot{\varepsilon \;},T\right)$. Many functions are used for the mathematical description of changes in value σ_p_ depending on the strain ε, temperature T, and strain rate $\dot{\varepsilon }$. One of them is the Hansel–Spittel Equation (1) [21]. This dependence is frequently used to derive value σ_p_ in computer programmes for numerical modelling of plastic working processes [22]:

(1) $$\sigma_{p}={\mathrm{Ae}}^{m_{1}T}\varepsilon^{m_{2}}{\dot{\varepsilon }}^{m_{3}}\varepsilon^{\frac{m_{4}}{\varepsilon }}{\left(1+\varepsilon \right)}^{m_{5}T}\varepsilon^{m_{7}T}{\dot{\varepsilon }}^{m_{8}T}T^{m_{9}}$$

where: σ**_p_** is the flow stress, ε is the true strain, $\dot{\varepsilon }$ is the strain rate, T is the temperature, m_1_–m_9_ are coefficients characterising magnesium alloys.

**Figure 1:**
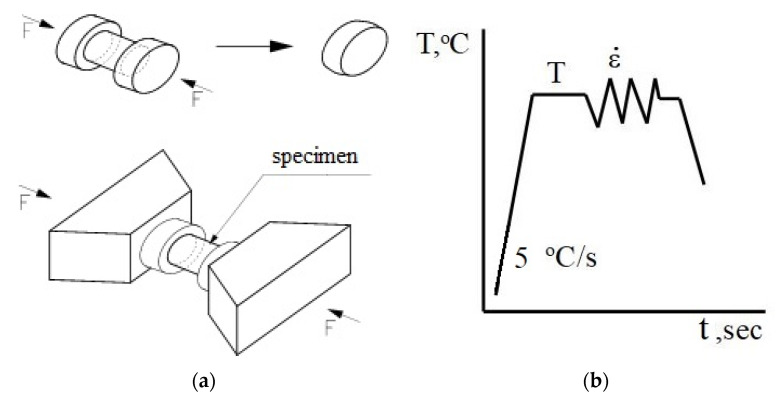
Geometry and dimension of load (**a**); and thermal cycle of physical simulation (**b**) [15].

In this paper, the rheological properties of the AZ91 magnesium alloy were determined on the basis of compression tests performed with a Gleeble 3800 metallurgical process simulator [22]. The Gleeble 3800 simulator makes it possible to carry out tests for a wide range of temperatures, corresponding to the actual conditions occurring in the analysed technological process [23]. Tests are carried out in a vacuum chamber at a constant temperature of the deformed sample. For plastometric tests, cylindrical samples with a diameter of 10 mm and a length of 12 mm are used (Figure 1a). The uniaxial compression test involves deformation of cylindrical samples between two well-lubricated planes. Given the ideal conditions, the compression of the samples should be isothermal. The area of interaction between the planes and the samples should have a zero value of the friction coefficient. Moreover, when the sample is compressed, no deformation consisting in losing its cylindrical shape should occur. Taking these conditions into account and assuming the invariability of the metal volume during the compression test, the determined dependencies of the actual stress on the actual deformation should be very similar to the conditions occurring in metal shaping in industrial processes.

The advantage of the uniaxial compression test at elevated temperature is that the data concerning the actual stress in relation to the actual deformation can be obtained for a much wider range of strains, compared to those tested, for example, in a tensile test.

The plastometric tests were conducted with the following parameters:

- Sample temperature: 200, 300, 400 °C,
- Strain rate: 0.1, 1, 10 s^−1^,
- True strain: max. 0.8.

The samples were heated at a constant rate of 5 °C/s, up to a preset temperature, held at this temperature for 20 s and then compressed [15]. The diagram for conducted tests is shown in Figure 1b.

**Figure 2:**
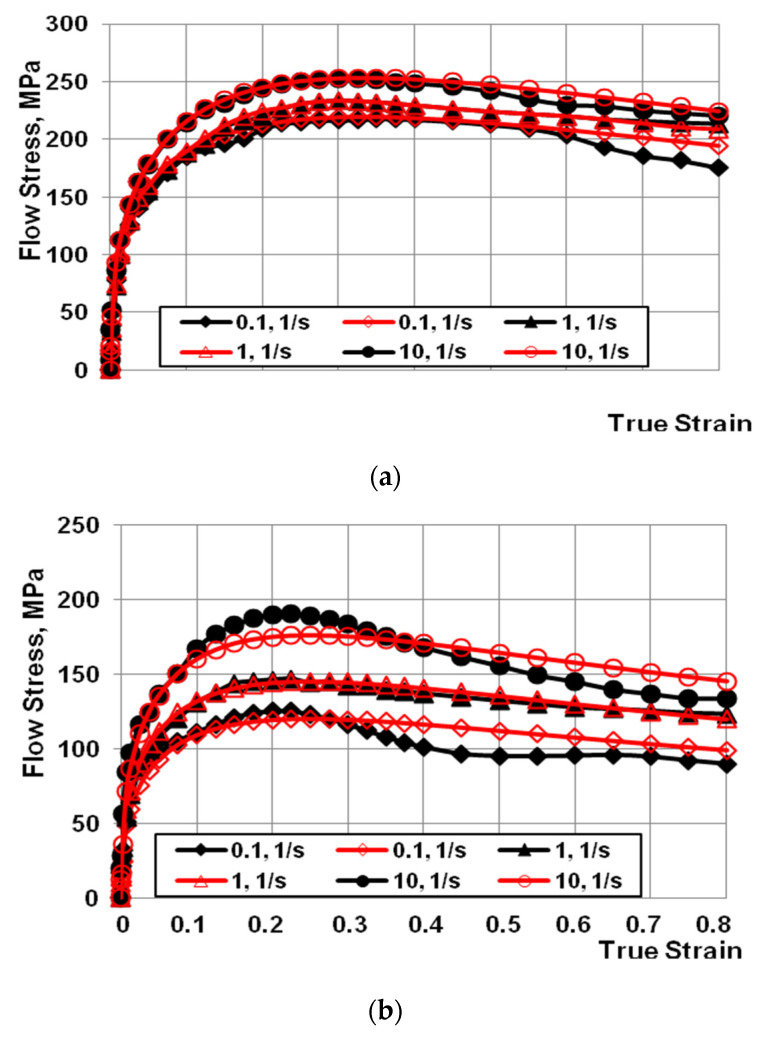

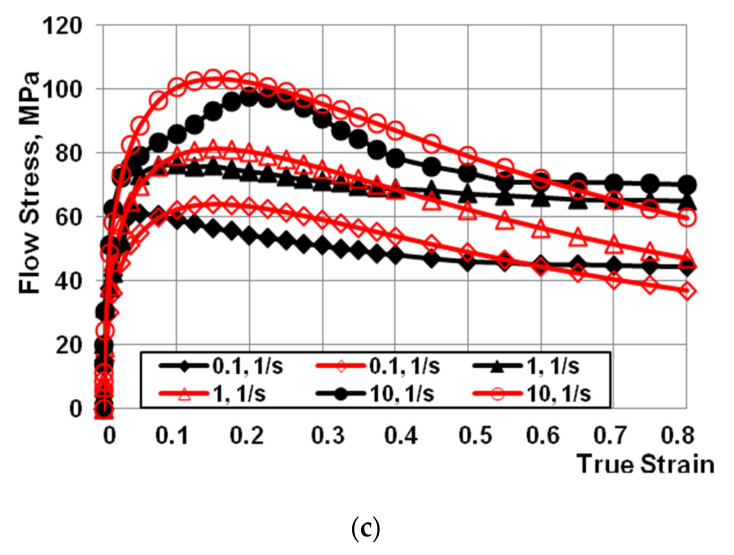
Work-hardening curves for the strain rate range of (0.1 s^−1^–10.0 s^−1^) at a temperature of: (**a**) 200, (**b**) 300, and (**c**) 400 °C. (Black indicates the experimental curves; red indicates the approximated curves) [15].

The dependencies between stress and actual deformation (Figure 2a,c) for the AZ91 magnesium alloy was developed on the basis of plastometric tests. The obtained test results were approximated in order to determine the coefficients of Equation (1) (Table 2).

Based on the data in Figure 2, it can be observed that, after reaching the critical deformation, the value of yield stress decreased with the increase of the actual deformation value, for the entire tested range of changes in temperature, deformation, and deformation rate. The decrease in the value of yield stress after reaching the maximum value is caused by the ongoing processes of dynamic healing. Dynamic processes of microstructure reconstruction are more intense at temperatures above 300 °C [15].

It is assumed that the coefficients of the approximating function (1) are sufficiently well-selected if the mean approximation error does not exceed 10%. After analysing the course of the actual curves and approximated curves presented in Figure 2, the approximation error was found to be less than 10%.

## 5. Research Methodology

**Figure 3:**
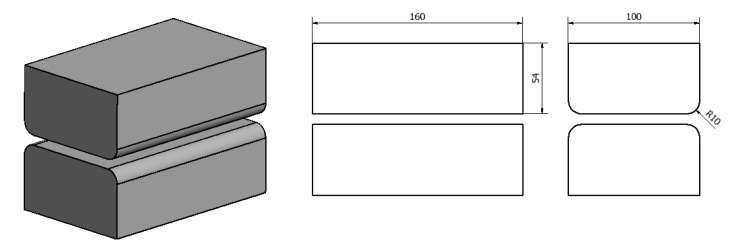
Shape and dimensions of flat dies used for AZ91 magnesium alloy forging [15].

**Figure 4:**
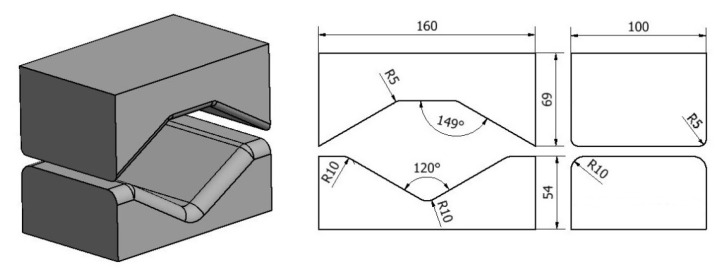
Shape and dimensions of rhombic-trapezoid dies used for AZ91 magnesium alloy forging [15].

The geometric shape and dimensions of the anvil deformation valley used to model the operation of elongation of an ingot made of an AZ91 magnesium alloy were selected on the basis of the literature and the authors’ own research carried out at the Department of Plastic Processing and Safety Engineering of the Czestochowa University of Technology [24,25,26,27,28,29,30,31,32,33] and are presented in Figure 3 and Figure 4.

The model charge for the numerical tests of the elongation process was an AZ91 magnesium alloy bar with diameter ϕ = 80 mm and length l = 80 mm. After the operations, the semi-finished product will have the following dimensions: 60 × 60 × 180. For numerical simulations, the number of nodes in the volume of the model charge was assumed to be 9116, while the number of tetrahedral elements was 41568.

Numerical modelling of the elongation operation process was performed using commercial software based on the Forge^®^NxT finite element method. This software allows for the thermomechanical simulation of, among others, plastic processing processes [34]. *Galerkin* equations were used for thermal calculations. The main technological parameters of the forging elongation operation were adopted on the basis of the authors’ own research [15,24,25,26,27,28] and the literature [29,30,31,32,33]. The initial conditions during the simulation of the elongation operation were the feed speed of the upper anvil equal to v = 8 mm/s, while the lower anvil was assumed to be stationary.

**Figure 5:**
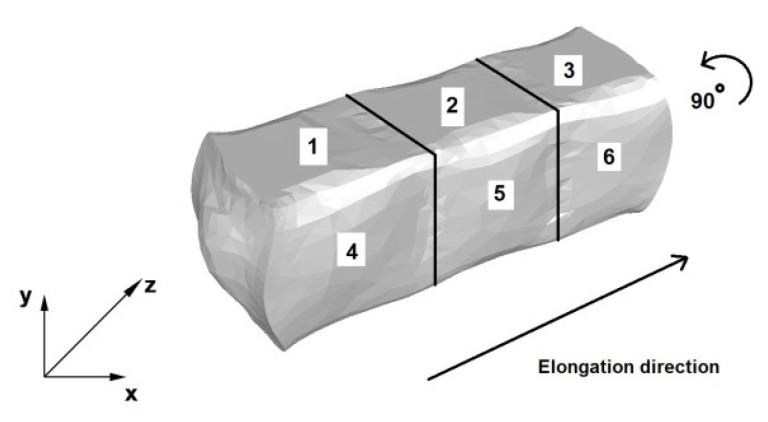
Flat die elongation process diagram [15].

Figure 5 shows a diagram of the operation of the AZ91 alloy rod elongation in flat anvils. Relative reduction of 40% was used in all forging passes in this operation. The forging passes were implemented in accordance with the forging direction indicated in Figure 5. The first three forging passes were carried out with a relative feed of 0.8, then the forging was rotated in the direction shown in Figure 5 by an angle of 90°, and then the anvils returned to the beginning of the forging (place of the Cartesian xyz coordinate system sketch). Later, three consecutive forging passes (strictly 4, 5, and 6) were performed, also with a density of 40% and a relative feed of 0.8.

**Figure 6:**
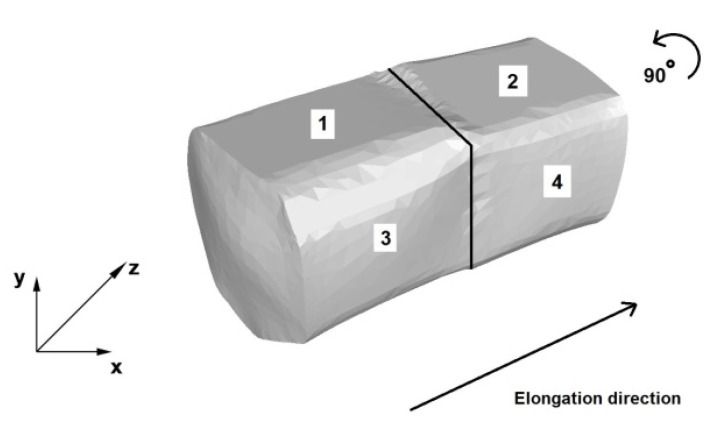
Trapezoid-rhombic die elongation operation diagram [15].

Figure 6 shows a diagram of the operation of the AZ91 alloy rod elongation in the trapezoid and diamond anvils. In the first two forging passes, one side of the bar was forged with a relative reduction of 25% and a relative feed of anvils of 0.8, in accordance with the direction shown in Figure 6. The forging was rotated, as shown in Figure 6, by an angle of 90°, and the anvils returned to the beginning of the forging (sketch of the Cartesian xyz coordinate system). Then, two successive forging passes (marked as 3 and 4) were carried out with a relative reduction of 40% and a relative feed of 0.8. The shape of the anvils determines the value of the applied relative reduction in the first forging passes.

In the paper, to simulate the elongation operation, a thermo-viscoplastic model of the deformed body, which is based on the theory of large plastic deformations, was used. The value of the friction coefficient adopted during the tests was μ = 0.3, in accordance with Coulomb’s law.

It was assumed that the heat transfer coefficient between the anvils and the material is λ = 5000 W/m^2^K, while the heat transfer coefficient between the metal and the environment is equal to λ = 10 W/m^2^K. The ambient temperature and the anvil temperature were assumed to be equal to 25 °C.

The initial temperature of the charge before the elongation operations in its entire volume was assumed to be the same and equal to 400 °C.

## 6. Analysis of the Distribution of Hydrostatic Pressure Values during the Elongation Operation

Figure 7, Figure 8, Figure 9, Figure 10, Figure 11, Figure 12, Figure 13 and Figure 14 show the distributions of hydrostatic pressure values obtained during numerical simulations of AZ91 magnesium alloy elongation in flat anvils.

**Figure 7:**
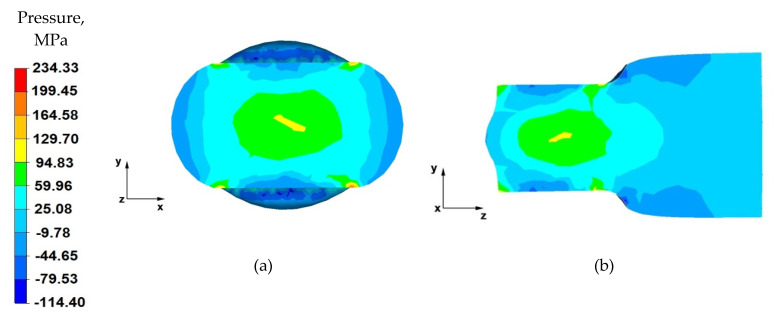
Distribution of the hydrostatic pressure of the AZ 91 alloy forging, deformed in the first pass with 40% reduction in flat anvils: (**a**) on the cross-section, (**b**) on the longitudinal section.

From the data in Figure 7a showing the distribution of hydrostatic pressure on the cross-section in the first stage of the elongation operation in flat anvils, it follows that, on the cross-section of the forging under consideration, except for the outer zones, there are high values of hydrostatic pressure, which means good plastic processing of the charge material. The pressure forces caused by the effect of the lower and upper anvils compensate parallelly to the y-axis, resulting in high-pressure values ranging from 130 to 60 MPa. This means that the material was forged through, and it should be assumed that, for almost the entire section of the forging, the degrees of forging will have high values, which is a desirable phenomenon in good practice of applying the elongation operation.

Based on the analysis of the data presented in Figure 7b, it can be concluded that, in the deformation valley, there is high hydrostatic pressure with values in the range 130–25 MPa, due to the pressure of the anvils. This proves good forging in the areas along the forging axis. Negative values of hydrostatic pressure (−10–−80 MPa) can be observed in the zones at the contact surface of the deformed forging with the working surfaces of the anvils. The cause of negative hydrostatic pressure values are the friction forces at the material–tool contact surfaces. This causes tensile stresses to occur in the near-surface layers of the forging. The occurrence of these stresses may cause delamination and cracking of the material.

**Figure 8:**
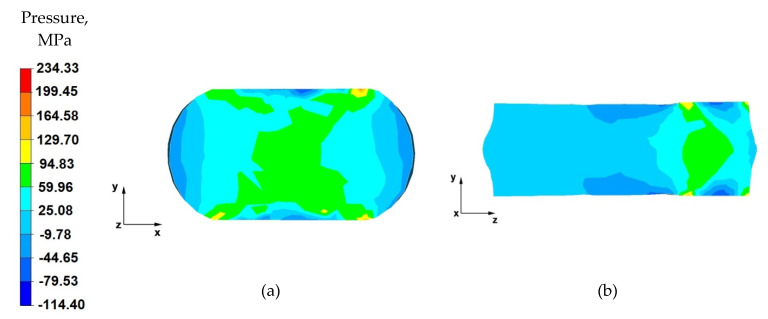
Distribution of the hydrostatic pressure of the AZ 91 alloy forging, deformed in the third pass with 40% reduction in flat anvils: (**a**) on the cross-section, (**b**) on the longitudinal section.

From the data shown in Figure 8a, it follows that a similar hydrostatic pressure distribution was obtained as in the first pass (Figure 7a). A forging cross is visible, which is characteristic for the elongation operation in flat anvils. It is undesirable when striving to obtain uniform distributions of the values of strains and stresses in the entire volume of the forging or in local zones, while it is desirable when striving to achieve the forging of the material lying in the zones including the forging cross. The high value of hydrostatic pressure inside the forging indicates a good degree of plastic processing of the material practically throughout its cross-section, with the exception of the outer zones of the forging.

By analysing the data contained in Figure 8b, it can be concluded that there are high values of hydrostatic pressure in the place of the anvil’s effect on the material. The pressure forces directed along the y-axis cause universal compression of the material, thus causing its plastic flow along the z-axis. The lack of hydrostatic pressure outside the deformation valley indicates the presence of high mean stress values with positive values, which causes the material to stretch.

**Figure 9:**
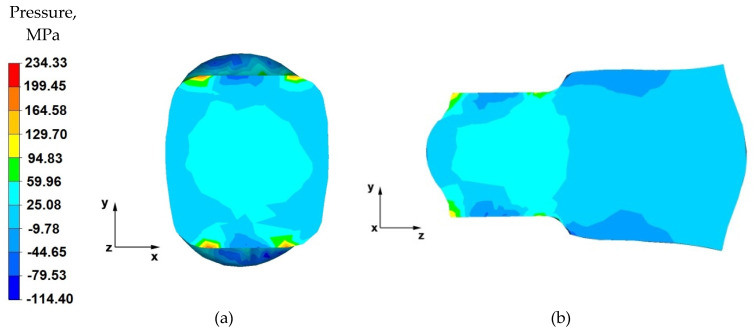
Distribution of the hydrostatic pressure of the AZ 91 alloy forging, deformed in the fourth pass with 40% reduction in flat anvils after slanting by 90°: (**a**) on the cross-section, (**b**) on the longitudinal section.

From the data shown in Figure 9a, it follows that a uniform distribution of hydrostatic pressure values in the considered section of the forging was obtained. However, the values of this pressure are half the values for the cases shown in Figure 7a and Figure 8a. The lower values of hydrostatic pressure are due to the use of a lower absolute reduction for this case. The obtained uniform distribution of hydrostatic pressure in the forging cross-section affects the achievement of uniform mechanical properties of the finished product.

Based on the data in Figure 9b, it can be stated that, in the deformation valley, the value of hydrostatic pressure is equal to 60 MPa, except for the areas located near the material–tool contact zones. For these areas, the hydrostatic pressure values were negative and ranged from −10 to −45 MPa. Outside the deformation valley, the pressure value was 25 MPa. Such a nature of the hydrostatic pressure value distribution proves good forcing through the material in the deformation valley.

**Figure 10:**
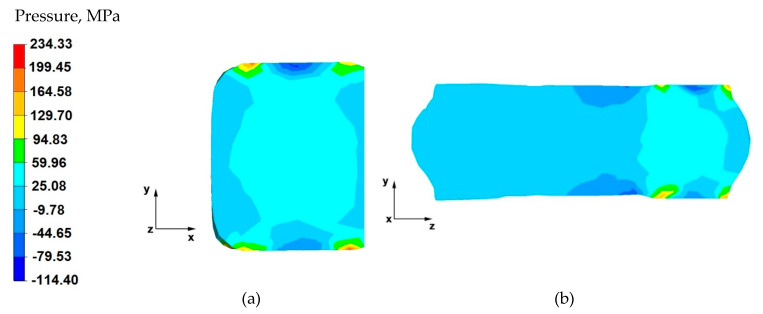
Distribution of the hydrostatic pressure of the AZ 91 alloy forging, deformed in the sixth pass with 40% reduction in flat anvils after slanting by 90°: (**a**) on the cross-section, (**b**) on the longitudinal section.

From the analysis of the data shown in Figure 10a, it follows that the forging cross formed during the deformation indicates good forging in the last deformation stage, ending the elongation operation of the AZ 91 alloy. Apart from the near-surface zone, which will be removed in the machining process, the hydrostatic pressure values fluctuated within the range of 60–25 MPa. Such good material processing in the last stage of the forging guarantees a homogeneous structure, and thus, appropriate mechanical properties.

By analysing the data presented in Figure 10b and obtained for the last forging pass in this elongation operation, it can be stated that the material was processed uniformly both in the zones under the action of the anvils, as well as in the entire longitudinal section along the *z*-axis. The values of hydrostatic pressure in this area of the forging ranged from 60 to 25 MPa. Slight local disturbances, which did not affect the processing of the material in the entire volume of the forging, occurred in the zones of contact of the material with the tool. It is extremely difficult to obtain, on the longitudinal section of the elongated forgings, identical and yet the same sign of the stress values in the entire volume, when carrying out the elongation operation in flat anvils.

**Figure 11:**
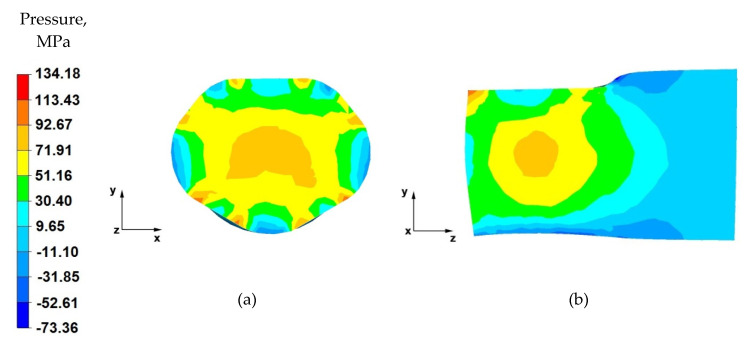
Distribution of the hydrostatic pressure of the AZ 91 alloy forging, deformed in the first pass with 25% reduction in trapezoid and diamond anvils: (**a**) on the cross-section, (**b**) on the longitudinal section.

From the data in Figure 11a, it follows that, after the first stage of the elongation operation in the shaped anvils, the appropriate forging of the forging was obtained, in accordance with good forging practice, except for the outer zones. Such a degree of plastic processing of the material was achieved through the variable geometry of the anvils used to elongate the alloy. Their working surfaces caused the directions of the pressure and friction forces from the variable geometry of the upper trapezoid and lower diamond anvils to cross. In almost the entire cross-section of the forgings, high values of hydrostatic pressure in the range of 93–30 MPa were obtained.

By analysing the data in Figure 11b, it can be observed that there are high values of hydrostatic pressure in the metal in the anvil action zones. The analysis of the hydrostatic pressure distribution on the longitudinal section of the forging shows that the AZ91 alloy core was forged through, which is very desirable in forging elongation operations, and not always achievable. There, the hydrostatic pressure values were within the range of 93–72~MPa. Good forging of the axial zone (in this case, along the *z*-axis) proves that good strength properties and good quality of the product made by hot elongation were obtained.

**Figure 12:**
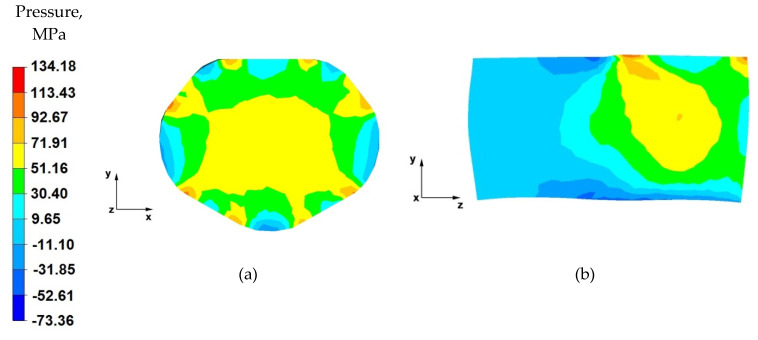
Distribution of the hydrostatic pressure of the AZ 91 alloy forging, deformed in the second pass with 25% reduction in trapezoid and diamond anvils: (**a**) on the cross-section, (**b**) on the longitudinal section.

Based on the data in Figure 12a, it can be concluded that the nature of the hydrostatic pressure fields is similar to that obtained in the first forging pass (Figure 8). Similarly to the first forging pass, a good material processing was obtained, from which it can be concluded that the finished product will be characterised by good mechanical properties.

By analysing the data in Figure 12b, it can be stated that, apart from the zones of contact of the material with the lower diamond anvil, the material flows freely without touching the top of the working surfaces of the anvil. Due to the lack of deformation resistance in this zone, tensile stresses occur there (no hydrostatic pressure). Apart from this small local part of the forging, the material has good plastic processing (the hydrostatic pressure value is within the range of 72–30 MPa), ensuring that it is forged through. In subsequent forging passes, after the forging is rotated by 90°, it will be important to maintain such a degree of processing.

**Figure 13:**
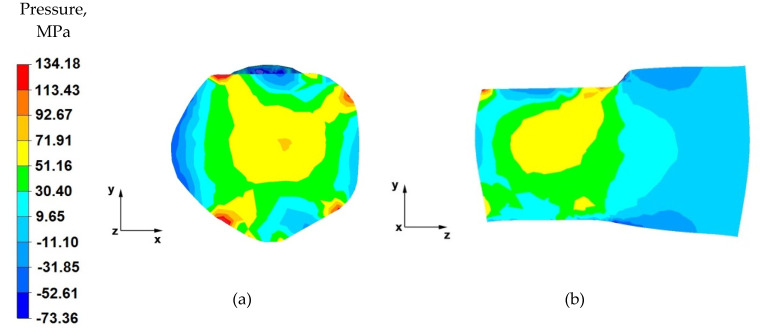
Distribution of the hydrostatic pressure of the AZ 91 alloy forging, deformed in the third pass with 40% reduction in trapezoid and diamond anvils after slanting by 90°: (**a**) on the cross-section, (**b**) on the longitudinal section.

Figure 13a shows the distribution of the hydrostatic pressure on the cross-section of the forging forged in the third pass with 40% reduction in trapezoid and diamond anvils after slanting by 90°. After the forging was rotated, it was possible to apply a relative reduction of 40%. The implementation of such deformation ensures the maintenance of high values of hydrostatic pressure in the range of 93–51 MPa in almost the entire volume of the forging. The only zones with lower values of hydrostatic pressure are the local outer zones. Local zones with lower values of hydrostatic pressure will be levelled in the next stages of forging. It is important that the material is forged well in the entire volume of the forging in the initial elongation operations.

From the analysis of the data in Figure 13b, it follows that an appropriate character of the stress distribution in the deformation valley, consistent with the good forging practice of conducting the elongation operation, was obtained. In the anvil interaction zone, the deformed metal showed high values of hydrostatic pressure (72–51 MPa).

**Figure 14:**
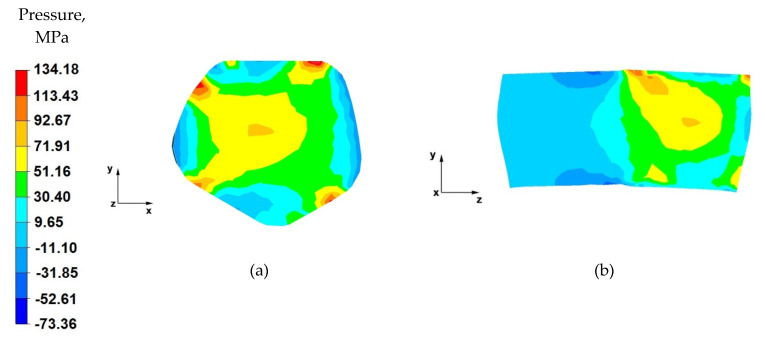
Distribution of the hydrostatic pressure of the AZ 91 alloy forging, deformed in the fourth pass with 40% reduction in trapezoid and diamond anvils after slanting by 90°: (**a**) on the cross-section, (**b**) on the longitudinal section.

Based on the data shown in Figure 14a, it can be concluded that, in the last step of the elongation operation, good plastic processing of the material was also obtained. The obtained values of hydrostatic pressure in the metal on its cross-section were within the range of 113–51 MPa. Obtaining the desired state of stresses is the result of the crossing in the forging core of the pressure force vectors, resulting from the effect of the side walls of the working surfaces of shaped anvils on the metal. Such a deformation method leads to the all-round compression state in the material located in the deformation valley.

By analysing the data in Figure 14b, it can be stated that, along the metal’s longitudinal section, there were high values of hydrostatic pressure, while outside the deformation valley, the value of hydrostatic pressure in the metal was small and amounted to about 10 MPa.

## 7. Analysis of the Effective Strain Distribution during the Elongation Operation

**Figure 15:**
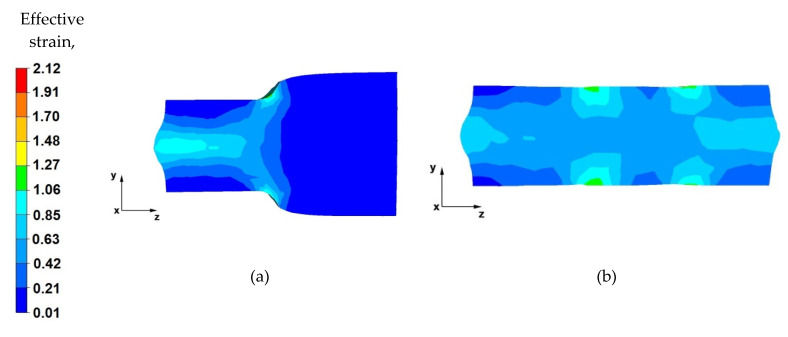
Distribution of the effective strain value on the longitudinal section of the AZ 91 alloy forging with 40% reduction in flat anvils (**a**) forged in the first pass, (**b**) forged in the third pass.

**Figure 16:**
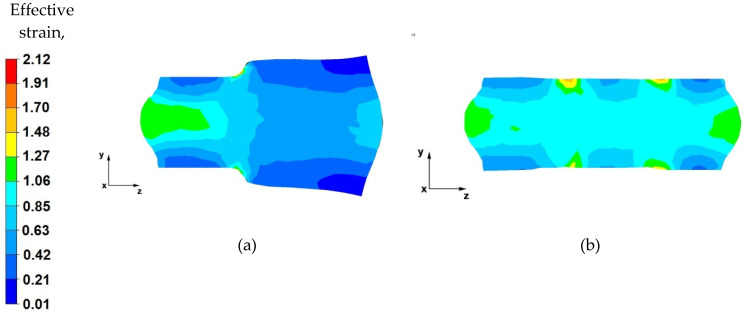
Distribution of the effective strain value on the longitudinal section of the AZ 91 forging, with 40% compression in flat anvils after slanting by an angle of 90°, (**a**) forged in the fourth pass, (**b**) forged in the sixth pass.

**Figure 17:**
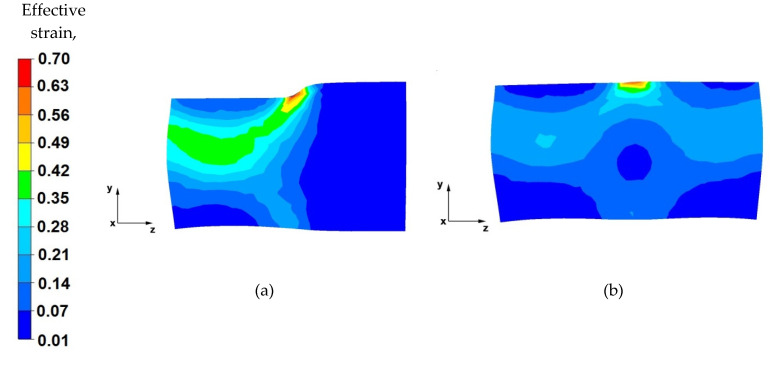
Distribution of the effective strain value on the longitudinal section of the AZ 91 alloy forging, with 25% reduction in trapezoid and diamond anvils, (**a**) forged in the first pass, (**b**) forged in the second pass.

**Figure 18:**
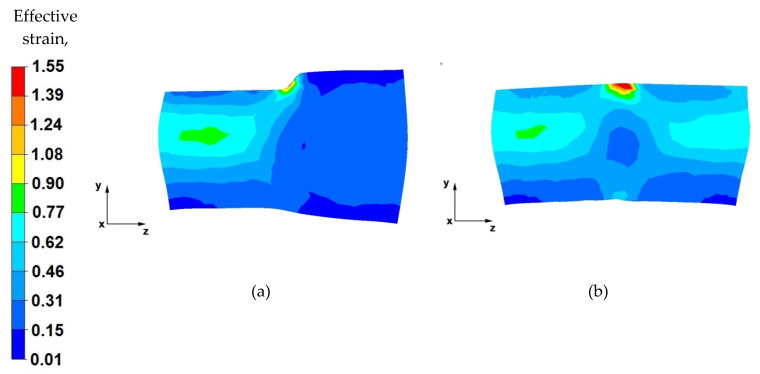
Distribution of the effective strain value on the longitudinal section of the AZ 91 forging, with 40% reduction in trapezoid and diamond anvils after slanting by 90°, (**a**) forged in the third pass, (**b**) forged in the fourth pass.

Figure 15, Figure 16, Figure 17 and Figure 18 show the distribution of the effective strain values obtained during the numerical modelling of the operation of extending the AZ91 magnesium alloy ingot in flat and shaped anvils.

By analysing the data in Figure 15a, it can be concluded that, in the deformation valley, in the areas along the longitudinal axis of the forging, the material was forged evenly, and there are strains of the highest value (1.06–0.85). Hence, the flow of metal is also greatest in the axial zone. It can be seen that the forging core in the deformation valley is uniformly forged, which is particularly desirable in the elongation operation stages when the temperature of the forging is close to the initial temperature, and the deformation resistance is relatively low. This means that the value of the relative reduction (40%) was selected rationally and in accordance with good forging practice. Lower values of the reduction would not result in such good forging through the core, and reductions above 40% would cause forging cracks in the axial zones. A large intensification of deformations is also visible in the places of the upper and lower anvils’ rounding, which is related to the deep penetration of the anvil side surfaces into the material. In the next stages of forging, after slanting, these places will be levelled.

Based on the analysis of the data contained in Figure 15b, it can be stated that the effective strain distribution on the longitudinal section, after the forging is completely forged to one side (i.e., before slanting), is even along its entire length. Uniform effective strain distribution proves the possibility of obtaining homogeneous mechanical properties of the finished product. Slight differences in the effective strain values occur in the places of successive relative anvil feeds. Otherwise, the subsurface zones will be removed in the subsequent machining steps.

By analysing the data shown in Figure 16a, it was observed that, after rotating the forging by an angle of 90°, the effective strain values in the forging core increased and fell within the range of 1.06–1.27. In the deformation valley, the material flows plastically along the *z*-axis in the opposite direction to the forging direction. The deformation intensity values in the zones under the direct influence of the anvils ranged from 0.63 to 0.42. Lower effective strain values on the contact surfaces of the material with the tools are a result of the higher deformation resistance caused by the friction forces occurring there.

From the data in Figure 16b, it can be concluded that there is a uniform effective strain distribution in the forged material. The occurrence of small areas with a higher effective strain value is related to the shifting of the anvils in the successive forging passes. Further areas of greater effective strain were observed in the front and rear parts of the forging, in the places of free metal flow along the *z*-axis, in the forging direction, and in the opposite direction. This metal flow pattern occurs in any elongation operation, especially in flat anvils. The uniform effective strain values distribution over the entire volume of the forging can be obtained by the appropriate selection of the relative anvil feed value. In the analysed operation of the AZ91 magnesium alloy ingot elongation, the relative feed was assumed to be 0.8.

Based on the data shown in Figure 17a, it can be stated that the effective strain value is unevenly distributed along the longitudinal section of the forged alloy. The reason for this condition is the use of a relative reduction of a low value of 25%, due to the geometric shapes of the anvils which, in the first stages of forming the forgings, before turning by 90°, make it impossible to use larger reductions. Additionally, the use of two different anvils results in asymmetric plastic flow of the metal in the deformation valley. The values of the pressure and friction forces from the upper trapezoid anvil were greater, and the deformation valley from the side of the trapezoid anvil was also greater. Therefore, an uneven distribution of the effective strain values can be observed (0.35–0.29). In the deformation valley, from the side of the lower trapezoid anvil, the deformation intensity value was 0.17–0.060. On the basis of the conducted analysis, it can be concluded that the obtained effective strain distribution is unfavourable and is not compliant with good forging practice of conducting the elongation operation. This unfavourable effective strain distribution will be eliminated in the subsequent forging passes.

From the data in Figure 17b, it follows that, after the second forging pass, a more even distribution of the effective strain values was obtained on the longitudinal section of the forging. The effective strain values ranged from 0.44 to 0.22. Low strain values were related to the low relative reduction of only 25% due to the contact of the side surfaces of the anvils, as well as different shape and dimensional parameters of the anvils used.

By analysing the effective strain distributions shown in Figure 18a, it can be observed that good forging occurred in its central part. Under the action of the anvils, the metal flows freely in the opposite direction to the *z*-axis, while the near-surface zones of the forging are less forged because the plastic flow of the metal in these zones is blocked by the side surfaces of both the diamond and trapezoid anvils. The most important thing in forging elongation operations is the appropriate forging of the axial zone, which is not always possible to achieve in other elongation operations. However, in the analysed case, it was possible to achieve this.

Based on the analysis of the data presented in Figure 18b, it can be concluded that, after successive metal forging passes in shaped anvils, the effective strain value is two times lower in the zones where there is no anvil interaction (0.31) than in the zones of their impact (0.77). The use of shaped anvils in the elongation operation of the anvil shape affects the uniform distribution of the effective strain in the anvil interaction zones, while it is the cause of the uneven distribution of the effective strain in the places of subsequent forging passes. However, in the entire volume of the deformed metal, these places are few, and they do not significantly affect the mechanical properties of the finished product.

## 8. Final Conclusions

Based on the analysis of the results of numerical modelling of the elongation operation of the AZ91 magnesium alloy ingot in flat and shaped anvils, the following final conclusions were formulated:

- From an economic and technological point of view, it is rational to carry out the elongation operation of the AZ91 magnesium alloy ingot in trapezoid and diamond shape anvils designed by the authors. As a result of the use of shaped anvils in the elongation operation, it was possible to reduce the number of forging passes from six to four, compared to the technology for which flat anvils were used, obtaining the same shape and dimensional parameters of the finished forging.
- During the elongation operation in both flat and shaped anvils, an even distribution of hydrostatic pressure was obtained. The hydrostatic pressure values were 94–59 MPa during the elongation operation in flat anvils and 91–51 MPa in the shaped anvils, which means good material processing.
- Negative values of hydrostatic pressure (no hydrostatic pressure) during the elongation operation in flat anvils can be observed on the contact surfaces of the deformed forging with the working surfaces of the anvils. On the other hand, during the elongation operation in diamond and trapezoid anvils, negative values of hydrostatic pressure occur on the surface of contact of the upper trapezoid anvil with the material. The cause of the negative hydrostatic pressure values is the friction forces occurring in the material–tool contact zones, which cause tensile stresses in the near-surface layers of the forging. The occurrence of these stresses may cause delamination and cracking of the material.
- The uniform distribution of the deformation intensity value during the elongation operation of the model AZ91 magnesium alloy ingot was obtained both in diamond and trapezoid and flat anvils. The deformation intensity values obtained in the shape anvils were lower compared to the values obtained in the flat anvils. Slight differences in the deformation intensity values occurred in the boundary points between successive relative anvil feeds. However, material from these zones is removed in subsequent machining steps.
- For deformation of the AZ91 magnesium alloy in hot forging operations, it is recommended to use shaped anvils in the initial stages of the forging formation, while flat anvils should be used in the final stages of forming. The homogeneity of the deformation intensity distribution was observed for the following technological parameters: relative reduction in the range of 25–40%, relative feed 0.8, forging rotation angle 90°. The elongation operation was carried out by forging on one side.

## Figures and Tables

**Table 1:** Chemical composition of the investigated alloy (%).

Alloy	Zn	Al	Si	Cu	Mn	Fe	Ni	Mg
AZ91	0.59	8.98	0.05	0.006	0.23	0.013	0.003	R

**Table 2:** Parameter values obtained from the approximation of Equation (1).

	A	m1	m2	m3	m4	m5	m7	m8	m9
AZ91	5985.99	−0.0116	0.37027	0.20062	−1.948 × 10^−5^	−0.008	0.5807	0.00021	2.54972

## Data Availability

Data sharing not applicable.
